# Real-time human-robot interaction and service provision using hybrid intelligent computing framework

**DOI:** 10.1371/journal.pone.0324986

**Published:** 2025-06-27

**Authors:** Mohammed Albekairi, Meshari D. Alanazi, Turki M. Alanazi, Mohamed Vall O. Mohamed, Khaled Kaaniche, Anis Sahbani, Ali Elrashidi

**Affiliations:** 1 Department of Electrical Engineering, College of Engineering, Jouf University, Sakakah, Saudi Arabia; 2 Department of Electrical Engineering, College of Engineering, University of Hafr Al Batin, Hafr Al Batin, Saudi Arabia; 3 Department of Computer Engineering and Networks, College of Computer and Information Sciences, Jouf University, Sakaka, KSA; 4 Enova Robotics S.A, Novation City Technopole, Sousse, Tunisia; 5 Institute for Intelligent Systems and Robotics (ISIR), CNRS, Sorbonne University, Paris, France; 6 Electrical Engineering Department, University of Business and Technology, Jeddah, Saudi Arabia; National Yang Ming Chiao Tung University, TAIWAN

## Abstract

Human-robot interaction has gained significant attention in various domains, including healthcare, customer service, and industrial automation. High computational cost, inefficient service matching, and elevated failure rates in dynamic service contexts are some primary disadvantages of existing query-processing systems. This research introduces a Hybrid Intelligent Computing Model (HICM) to improve robots’ ability to process inquiries autonomously. The goal is to make robots better at responding to human questions in real time with efficient, personalized, and context-specific solutions. Using self-organized computing approaches, robotic agents can reliably provide end-users with services suited to their demands. Due to their autonomous nature, robots must be able to calculate quickly and accurately to provide timely services. To meet these needs, the proposed HICM incorporates a sophisticated decision-support system to handle human questions and find the appropriate services. Within this decision-making framework, the model evaluates the characteristics and relevance of questions about accessible services by combining annealing and Tabu Search approaches. To avoid addressing queries incompatibly, the Tabu Search technique approaches query resolution as a non-convergent optimization issue. Comparing HICM’s performance to other models reveals significant improvements over CDS, DGTA, and CCS. In particular, HICM reduced calculation time by 8.67%, service time by 15.09%, and failure rates by 7.87%. In terms of important metrics, HICM fared better than the competing models. Its success factor was 11.8% higher, its matching ratio was 14.88% higher, and its failure rates were 6.22% lower. These findings demonstrate the model’s efficiency and reliability in terms of robotic query processing and real-time service delivery.

## 1. Introduction

### 1.1. Background

The increasing demand for real-time collaboration, particularly in critical situations in robotics systems, necessitates the practical application of context-aware platforms and the compatibility of existing IT/OT technologies to effectively implement edge computing and fog computing concepts [1]. Robotic communications and interactive prototypes facilitate the provision of autonomous service responses [2]. Robot-based services provide pervasive interaction support and user responses in different real-time applications. In such interactions, the robots rely on user inputs in the form of queries [3]. Being the most comprehensive and popular cloud platform globally, Amazon Web Services (AWS) offers more than 200 fully operational services from data centers worldwide. Many customers utilize AWS to lower expenses, boost flexibility, and speed progress, including major companies, successful government entities, and rapidly expanding new businesses [4]. The computing resources of robotic applications rely on heterogeneous networks, outward communication, cloud systems, and the prominent Internet of Things (IoT). Such an interconnected environment helps meet user requirements regardless of the type of application demand and interaction session time [5].

A real-time augmented reality system must accomplish many things, such as receiving images from input sensors, processing them, and then providing the user with the correct responses. Computer vision, robotics, and image processing have all benefited from researchers’ increased access to storage and processing power [6]. The cloud platform integrates many communication technologies and service providers to offer various services. Additionally, it offers fault-tolerant robot interaction assistance using distributed and decentralized services. [7]. A humanoid robot that can identify and express different emotions would be incredibly helpful for service jobs requiring a warm and inviting demeanor. [8]. Teleoperation between robots and people in hazardous or inconvenient locations is a growing trend in the automation and robotics industry. The delay between issuing orders and their execution is a persistent issue in this field; it reduces operator awareness, performance, and mental effort [9]. No cutting-edge processing power can eliminate these delays, particularly problematic for long-distance tasks. The majority of current approaches to dealing with these delays include the use of machines. However, using human perceptions to enhance the subjective teleoperation experience is still in infancy [10].

The computing techniques designed for robot-aided applications require portability and offloading features. Different computing features, such as scalability, adaptability, and concurrency, are prominent in determining the service reliability of robot services [11]. Various contemporary robotic uses gain advantages from centralized thinking and processing systems. However, modern advanced robotic systems can produce a substantial amount of data, potentially surpassing the capacity of existing wireless communication systems if they transmit all data without further consideration [12]. The distributed nature of the computing models provides less complexity in managing user queries and service responses. Based on the application demands, we model a varying density of computing and service resources for robotic interaction [13]. Hybrid computing and optimization solutions simplify the complexity of processing and query handling to prevent response failures. Furthermore, the application gains a precise computation and query processing feature by comprehending the user’s requirements based on the query. Combining different computing methods and techniques helps to provide reliable assistance in robot query processing and service endowments [14]. Accurate and adaptable query processing is essential for human-robot interaction in real-time service applications. This will improve the user experience and operational efficiency. Disruptions to smooth robotic decision-making are caused by current frameworks’ inadequate response times, ineffective query-to-service mapping, and elevated failure rates. Especially in situations when resources are limited and conditions are constantly changing, these constraints make autonomous service systems less reliable and less scalable [15]. This work analyzes the computational limits and performance bottlenecks in existing models to examine these issues. According to the results, a more effective, fast, accurate, and flexible query processing method is needed. This study addresses a gap in the literature by identifying the most critical optimization parameters for robotic decision-making, thereby enhancing human-robot interactions through the provision of real-time, dependable, and context-aware services.

### 1.2. Problem statement

Existing models of real-time human-robot interaction incur significant computing overhead, fail to accurately map service queries, and exhibit higher failure rates in dynamic contexts; these are the primary issues this study aims to address. Delays and mismatches in robotic decision-making result from traditional systems’ failure to maximize service selection while preserving responsiveness. The task is to design a smart computing architecture that can quickly handle queries, reduce service outages, and improve decision accuracy while maintaining high computational efficiency. To incorporate intelligent search methods that balance speed, flexibility, and accuracy in query resolution, this research formulates the issue as an optimization assignment. To ensure scalable, dependable, and context-aware service delivery, the project aims to develop a more effective method for processing robotic queries.

The problem addressed in this paper is presented as a combinatorial optimization problem, where the aim is to find the optimal solution $S^{*}\in \;S$ from a finite set of possible solutions 𝒮, such that the objective function f(S) is minimized. Mathematically, the goal can be stated as: $S^{*}=\mathrm{\backslash arg}\max_{S\in 𝒮}f(S).$ Here, S is a candidate solution and f(S) estimates its quality based on cost, run time, energy usage, etc., The form of every solution S must meet all the constraints of a problem, which are included in the definition of the feasible set  S. The objective function is the most important factor guiding the hybrid Tabu Search–Simulated Annealing algorithm, as it provides the direction of the search and the acceptance rule at any iteration.

### 1.3. Motivation

Research in this area is motivated by the growing need for cognitive human-robot interaction in many fields, such as healthcare, customer service, and industrial automation. However, existing models have difficulty adapting to ever-changing environments due to inefficient query-to-service mapping, excessive computational overhead, and higher failure rates. With the increasing importance of robots in autonomous decision-making, a computing framework that improves the accuracy of query processing while keeping computational efficiency high is urgently needed. To compensate for it, our research is working on a model to boost system reliability, decrease service mismatches, and speed up reaction times. Through resolving these issues, the study advances the state of the art in intelligent robotic systems that can provide contextually aware and individually tailored services in practical settings.

### 1.4. Novelty

The novel aspect of HICM is its ability to adaptively combine Tabu Search and Simulated Annealing to handle dynamic, high-dimensional service optimization problems in real-time robotic query processing. Using Tabu Search, HICM can maintain a non-convergent optimization framework, guaranteeing ongoing query refinement and preventing service allocation mismatches, unlike traditional scheduling or logistics systems. Tabu Search utilizes improved solutions by constantly updating a memory-based limitation list, whereas Simulated Annealing enhances exploration by probabilistically accepting suboptimal solutions. Outperforming current models, such as CDS, DGTA, and CCS, in terms of computational efficiency and response accuracy, this hybrid technique offers scalable, self-organizing computing that adapts to dynamic user interactions. Differentiating from more conventional implementations in other fields, HICM delivers better query-service matching by combining these approaches into a real-time, autonomous robotic framework. This results in reduced service times and lower failure rates while maintaining high flexibility.

While newer algorithms, such as Particle Swarm Optimization (PSO), Differential Evolution (DE), Genetic Algorithms (GA), and reinforcement learning, have been proven highly promising, our selection of Simulated Annealing (SA) is cautious, given its simplicity, strength, and high capacity to escape local optima. With Tabu Search (TS) added, the hybrid Tabu Search – Simulated Annealing (TS-SA) method has a fair balance between exploitation and exploration. Our aim is to demonstrate that even older algorithms, when well-hybridized, remain competitive. Later research can explore the inclusion of or benchmarking against more advanced optimization methods.

The main contributions of the study are:

- The HICM is proposed to enhance robots’ autonomous query processing capabilities and facilitate personalized service delivery in real-time through self-organized computing techniques.
- An advanced hybrid algorithmic approach is aimed to be developed, combining annealing and Tabu Search techniques to optimize query processing and service matching while incorporating self-adjustment features to address situations where convergence is inadequate.
- A comprehensive assessment system is intended to evaluate the model’s efficiency in handling autonomous queries and interacting with humans.

This paper is structured as follows: Section 2 reviews related work. Section 3 presents the proposed HICM, which integrates Tabu Search and Simulated Annealing in detail. Section 4 provides a comprehensive performance evaluation, and Section 5 concludes the study, highlighting potential directions for future research.

## 2. Related works

Bhatta, K., & Chang, Q [16] proposed a flexible manufacturing system (FMS) that is operated by mobile, multiskilled robots, taking into consideration a model that unifies robots, distinct workstation processes, and product quality, utilizing a heterogeneous graph structure. Heterogeneous Graph Neural Networks (HGNNs) generate node embeddings that reflect global information by aggregating local information from various nodes in graph models.

Chiang, B. [17] suggested a smartphone app that uses Bluetooth inertial measurement units (IMUs) and a smartphone to record human motion. According to the study, the optimal ModelView-ViewModel architecture will preserve performance and ensure effective app data exchange while offering the greatest user interface. The program was created using Apple’s integrated programming environment (IDE), Xcode, and was intended to run natively on iOS. In an unconventional method for user data collection, this application collects feedback from users both positive and negative—to improve the application.

Zhou et al. [18] provided a broad overview of the future interactions between the automotive, transportation, and metaverse industries by outlining the framework of Vetaverse. Through the description of the Metaverse framework and the analysis of key enabler technologies, we unveil this emerging trend. The author also looks at open questions and possible directions for further research while showcasing some fascinating Vetaverse offerings. A cloud robot is introduced in [19] to improve the effectiveness of both the integrated and content approaches. Three aspects contribute to developing a context-aware dialoguing (CDS) service; the first is the training phase, which delivers the response to the user. The system extracts data from external knowledge and subsequently integrates contextual information and dialogual content. Mohd Taib et al. [20] significantly contributed to our understanding of advisory systems, specifically in the advisory structure’s use of artificial intelligence, methods, and validation process. Ultimately, this review contributes to a more comprehensive understanding of advisory systems. A distributed game theory algorithm (DGTA) is developed to address multiple heterogeneous cloud robot computing offloading. In [21], partial offloading is used to evaluate the task to achieve Nash equilibrium. The scope of this work is to reduce the completion time and improve service quality in the cloud environment.

A deep belief network (DBN), multilayer perceptron (MLP), cognitive computing system (CCS) model, and linear perceptron (DBNLP) are developed in [22]. Here, the objective is to enhance accuracy and ensure the safety of the robots. This approach reduces time consumption and optimizes resource allocation. Zheng et al. [23] emphasized the need to understand the mechanics underlying the capacity of spiking neural networks for learning, while discussing the potential of these networks to process temporal information. Zheng et al. [23] present a multicompartment spike neuronal model with longitudinal dendritic heterogeneity to represent components over several timescales. Experiments show that the model can handle difficult temporal calculations and do better than regular spiking networks of neurons on several recognition tests. This suggests that neuromorphic computing could be useful in real life.

Zahabi, M., & Park, J. [24] The proposed computational models, called cognitive performance models (CPM), simulate how people behave while interacting with interfaces and offer details on user intents and information processing. These models can distinguish serial and parallel processes, predict operator job performance and cognitive workload, and perform in-depth task analysis. Liu et al. [25] explored the role of robotic equipment in smart manufacturing, highlighting its benefits in productivity and human workload relief. It covers the background of smart robotic manufacturing, including definitions, categories, and leading technologies like imitation learning, policy gradient learning, value function learning, actor-critic learning, and model-based learning. It also discusses training tools, benchmarks, and comparisons among different robot learning methods. The review discusses typical industrial robotic grasping, assembly, process control, and human-robot collaboration applications. It concludes with a summary of open problems and future research directions.

Azam et al. [26] proposed a thorough analysis of intrusion detection methods, the most recent IDS taxonomy, and widely used evaluation datasets. To improve network security, it addresses attacker evasion tactics and the difficulties in countering them. It highlights research roadblocks and suggests a paradigm for future studies to solve methodological flaws. A methodology for identifying result anomalies is suggested using the decision tree, renowned for its speed and ease of use, by merging the results of a comparison survey. Kraus et al. [27] showed that artificial intelligence could digitize business processes in enterprises to support Industry 4.0 and the search for socio-economic progress in the digital ecosystem and digital entrepreneurship. It discusses AI’s positive and negative effects on digital infrastructure and recommends ways to apply it. The fourth wave of artificial intelligence development is predicted to provide computer intelligence that understands and alters the world, benefiting highly organized surroundings and other human activities. The study examines how technical capabilities are used to form Industry 4.0 digital enterprises and proposes ways to develop artificial intelligence technologies, such as tools for users to simplify AI configuration and act without developers.

Ettalibi et al. [28] proposed Computer Vision (CV) and Machine Vision (MV) technologies that enable computers to recognize and interpret images and videos for a range of applications, including industrial quality control and medical diagnostics. It emphasizes the use of basic color identification techniques and artificial intelligence tools, such as machine learning and deep learning. Real-time quality control through industrial CV applications is examined, along with an analysis of the results, suggestions, and directions for further research.

Zhou et al. [29] proposed a novel approach to real-time reactive handling of deformable linear items in human-robot cooperation, which is presented in this work. It combines a fixed-time sliding controller with a topological latent representation for smooth interaction. For better performance, the model captures the dynamism of object shape. Experiments involving human-robot interactions and motor-robot simulations verify the efficacy of the strategy. In [30], it is demonstrated that Human-Robot Interaction (HRI) facilitates the detection of objects in real-time. Projection misidentification can cause recognition mistakes. In projection-dependent input processing (PDIP), technology reduces misidentifications in object recognition. The labeled analysis separates conjoined indices from the all-dimensional visualization image. To avoid errors, dimension projections identify non-correlating indices. Using labels to match inputs to stored inputs prevents plane and index matching errors. Measurements like recognition ratio, time, complexity, and error verify the approach.

Abdullah Ayub Khan et al. [31] suggested the blockchain distributed ledger technology for the Internet of Medical Things (BDLT-IoMT). This suggested architecture offers a new interoperable method by fixing the three major issues: data integrity, node-to-node communication, and infrastructure security. Results based on simulations show that the method is novel; there are large differences of 1.37%,1.56%, and 1.87%, respectively. Ensuring the safety of automated decision-making via infrastructure security; (ii) Maintaining the integrity of data sharing and exchange, and (iii) Optimizing network resources to facilitate communication across diverse devices. These three areas set the stage for the assessment. Abdullah Ayub Khan et al. [32] proposed the Blockchain Augmented Intelligence of Things for enterprise management (BAIoT-EMS). The platform’s consortium network and InterPlanetary File Storage (IPFS) provide secure storage and transaction management. Smart contracts are used to automate and protect activities such as device registration. A new multi-proof-of-work consensus approach is used for efficient analysis, validation, and verification of AIoT transactions. The simulation results demonstrate that the proposed framework is practical, improving performance by 63.51% and reducing computational power consumption by 11.75%.

Abdullah Ayub Khan et al. [33] recommended the Blockchain-enabled infrastructural security solution for serverless consortium fog and edge computing. The primary goal of this article is to organize survey reports and newly published studies on the state of the art in edge-distributed computing and outsourced computing, including fog and edge computing. Furthermore, we introduce a distributed, outsourced compute architecture that is serverless, edge-based, and powered by blockchain technology and Hyperledger Sawtooth. This theory-based method provides strong data security concerning transparency, privacy, integrity, and provenance when storing outsourced computational ledgers in immutable storage. The paper outlines how the proposed taxonomy differs from the current system in several key areas, including privacy and security. The list concludes with a few limits and outstanding research difficulties, but it does point to several potential paths for future study.

Abdullah Ayub Khan et al. [34] presented Digital Forensics for the Socio-Cyber World (DF-SCW). Detecting and analyzing deep false media investigations on social media platforms is possible in an AI-enabled socio-cyber environment, particularly one that uses deep neural networks (DNNs). To determine details about the original, it looks for nearby pixels in the same medium (such as pictures or films) and compares their values. To prevent harmful and harmful efforts, such as a strong leader announcing war, there is a media flag. Marking these postings as phony encourages digital investigators not to share them. Furthermore, this study can enhance the digital forensics ecosystem, enabling more accurate qualitative judgments in real-time when social media users post videos. Three distinct platforms, including Instagram, Facebook, and Twitter, are used to evaluate the simulation of the proposed DF-SCW. When detecting, identifying, and analyzing deepfake material, the DF-SCW fared better by a margin of 3.77% in the experiment.

Abdullah Ayub Khan et al. [35] introduced the Open Radio Access Network Architecture With Machine Learning for Beyond 5G in Industrial 5.0 (ORAN-B5G). Based on machine learning, the primary objective of this intelligent system is load balancing. It achieves this by utilizing metaheuristic optimization techniques, including those provided by Artificial Neural Networks and Particle Swarm Optimization, to meet the demands of the telecommunications sector, such as ensuring product compatibility. The author automates transactional load allocation across multiple linked units utilizing third-generation partnership project standards, increasing the proposed system’s dependability. Within the hierarchy of AI-enabled automation lies this intelligent system. Conversely, next-generation intercommunication hurdles, such as those after 5G, are investigated by AI-enabled open radio access control. Privacy and security, deterministic latency and capabilities, privacy and security testing, and controller architectures based on artificial intelligence are all covered.

Abdullah Ayub Khan et al. [36] discussed the Secure Remote Sensing Data With Blockchain Distributed Ledger Technology (DLT). This article utilizes Partial Swarm Optimization (PSO) and a secure blockchain-based distributed consortium network to develop an optimal data delivery method for the smart cities analytical model. This study provides three new insights. First, it provides a secure way to send data by integrating blockchain technology with machine learning to determine the most efficient method for transmitting data across encrypted channels. Secondly, NuCypher proxy re-encryption-enabled value encryption, a public key cryptographic method that does not require cipher conversion, is used to carry out neighborhood encryption sequences. Thirdly, by improving record management and preservation, Artificial Neural Networks (ANNs) can address the issue of categorizing data delivery in smart cities.

Abdullah Ayub Khan et al. [37] deliberated the Blockchain lightweight Proof-of-Elapsed Time (B-LPoET) for efficient distributed transaction execution and security. Disputes arise over how the preset PoET can be enhanced to become a lightweight PoET in areas such as transaction verification, block creation, mining rights, and participant identification before joining. By implementing a lightweight topology for node selection and reducing waiting time through multithreading, the B-LPoET architecture improves the cost-effectiveness of multi-node efficiency and enhances the system’s scalability. Compared to the current PoET and other state-of-the-art consensus, the experimental findings show that the B-LPoET performs better.

Motivated by the social interactions that occur within groups and the influence that leaders have on their followers, Rahman, C. M. [38] presented a new metaheuristic known as the Group Learning Algorithm. The exploration and exploitation phases are integrated to optimize challenging activities. When tested against both the traditional and CEC-C06 2019 benchmarks, the algorithm consistently performed better than the others, showcasing its powerful optimization capabilities. Despite the encouraging results, further testing in various areas is necessary to assess scalability and generalizability.

Damasevicius, R. [39] proposed a framework for analyzing common patterns in heuristic optimization algorithms, encompassing initialization, local search, diversity maintenance, adaptation, and stochasticity. Techniques include case studies of current algorithms and literature reviews. Results show that these patterns can direct the development of new heuristics and have a considerable impact on algorithm performance. Nevertheless, the study is mainly qualitative, and the lack of quantitative validation across many issue areas still prevents wider use.

Xu et al. [40] proposed REINFORCE-OPT, a novel iterative optimization technique that utilizes reinforcement learning to address inverse, stochastic, and complex problems. The method uses an algorithm akin to REINFORCE to parameterize and update solution search rules. Results from experiments demonstrate better performance than conventional techniques, particularly in addressing initial value sensitivity and escaping local optima. Nevertheless, the algorithm’s convergence is limited, and its effectiveness may vary depending on the type of task or probability model.

Due to excessive computing overhead, imprecise query-to-service mapping, and increasing failure rates, existing robotic query-processing models, such as CDS, DGTA, and CCS, demonstrate inefficiencies in real-time human-robot interaction. Delays and mismatches in service execution result from less-than-ideal decision-making techniques that struggle to adapt to changing service contexts. The primary challenge is to develop an AI-powered system that can optimize query resolution while maintaining computational efficiency.

## 3. Proposed work

### 3.1 Robot interaction with HICM architecture

**Fig 1:**
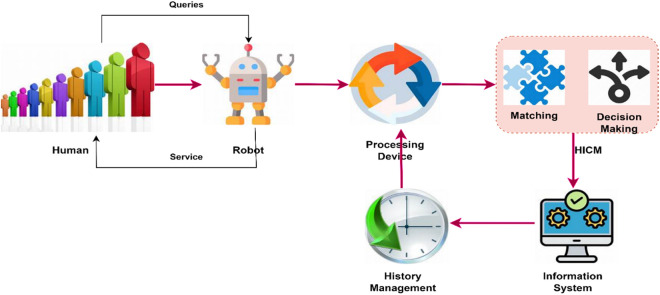
Proposed HICM in Robot-Interaction Services.

We use a real-time robot to enhance their interaction by sharing and retrieving information. This paper introduces HICM, a method for reliably processing human queries and retrieving services. Since it is a hybrid method, it employs two types of processing, namely Tabu Search and Simulated Annealing, to minimize service failures for the end user. Fig 1 illustrates the architecture of robot interaction with HICM. Table 1 shows the Nomenclature.

**Table 1:** Nomenclature.

Variables	Description
$q_{u}$	Query
$k_{0}$	Detection
$r_{s}andr_{r}$	sender and receiver robot
$p^{\prime }$	Response
$\left\{s_{0},s_{1},\ldots s_{n}\right\}$	number of services
$t_{e}$	allocated time
${\mathbb{Q}}^{\prime }$	query processing
$\alpha^{\prime }$	Tabu Search
$b_{0}$	current services
z	neighboring states
${\;W}_{0}$	tabu list
$x_{0}$	moves analyzed
${𝕞}^{\prime }$	modification of service
ρ	neighborhood balanced
${ℋ}^{\prime }$	History
$\vartheta^{\prime }$	Intensification
$\zeta_{0}$	Diversification
$g_{0}$	Verification
${𝒹}^{\prime }$	decision-making
${\;E}_{0}$	Simulated Annealing

This work aims to enhance both the success rate and the matching ratio while reducing the number of iteration steps within a given time slot. In equation (1), the requested user and end-user are defined to establish a communication link; here, the users interact with robots.


$$k_{0}=\sum_{r_{s}}^{q_{u}}s_{0}+t_{e}*\left(\frac{\Upsilon^{\prime }}{\frac{q_{u}}{s_{k}}}\right)+\left(\frac{\prod j_{0}-t_{e}}{q_{u}*p^{\prime }}\right)*\left(\prod_{v_{c}}\left(r_{r}+\Upsilon^{\prime }\left(s_{0}\right)-r_{s}\right)+\left(\frac{s_{0}-j_{0}}{m_{i}}\right)\right)*\varpi +\left(\Upsilon_{0}-\Upsilon^{\prime }\right)+\left({𝕊}_{k}-t_{e}\right)$$ (1)

The equation (1) identifies the communication link between the sender robot and the receiver robot, also referred to as the end-user, where the latter receives the input in the form of a query, represented as $q_{u}$. The detection is derived as $k_{0}$ where the sender and receiver robot are defined as $r_{s}andr_{r}$. In this case, the request is forwarded to the end-user, and the seeking of services is processed and provides the response termed $p^{\prime }$. Here, the number of services is processed and defined as the set $\{s_{0},s_{1},...,s_{n}\},$ where each$s_{i}$ represents an individual service instance. The total number of services is (n+1), and they are executed within an allocated time slot denoted by $t_{e}$.

To achieve a balance between exploration and exploitation in real-time query processing, HICM’s integration of Tabu Search and Simulated Annealing is crucial. This guarantees accurate and adaptive service matching. By accepting less-than-ideal answers probabilistically early and avoiding premature convergence, Simulated Annealing allows for extensive exploration of the search space. Alternatively, Tabu Search guides optimization away from local optima and refines the answer by maintaining a dynamic memory structure that prevents the return of previously investigated states. Whereas traditional single-method techniques suffer from excessive randomness or stagnation when faced with dynamic constraints, this synergy ensures that query-service mapping remains efficient. By combining the two methods, HICM can reduce reaction time and failure rates in autonomous robotic interactions while maintaining flexibility, thanks to its higher computational efficiency.

### 3.2 Query processing and service matching mechanism

In this processing, query matching is performed to retrieve the services for the end-user. The matching and mismatching is denoted as $\Upsilon^{\prime }and\Upsilon_{0}$, and the seeking is evaluated that is represented as ${𝕊}_{k}$ whereas convergences are denoted asϖ. From the acquired query, the similarity of services is derived as $m_{i}$ to the end-user. Thus, communication between the sender and receiver robots is established for the requested services. Here, communication is represented as $\chi_{0}$ where the similar matching of services is retrieved in an allocated time slot; in the above equation, it fetches the input as the query from the robot, and the output is the communication link. The query processing is derived by formulating the below equation (2), where the output of equation (1) is provided as the input for this method, and the seeking of similarity is the result of this equation.


$${\mathbb{Q}}^{\prime }=\begin{array}{c} \prod_{k_{0}}\left(s_{0}*r_{s}\right)+\left(\frac{q_{u}*m_{i}}{{𝕊}_{k}}\right)-\left(p^{\prime }+v_{c}\right)\\ \left(\frac{\left[\frac{r_{r}}{r_{s}}+q_{u}-p^{\prime }\right]}{\chi_{0}}\right)*\sum_{{𝕊}_{k}}^{m_{i}}\left(s_{0}*v_{c}\right)+\left(p^{\prime }-t_{e}\right) \end{array}\}$$ (2)

In the above equation (2), the input is fetched from the output of the communication link and yields the matching and mismatching processes. The query processing is represented as $Q^{\prime },$ where the robot requests for the services. The request derives the relevant service as the response to the end-user. This is formulated as $\left(\frac{q_{u}*m_{i}}{{𝕊}_{k}}\right)-\left(p^{\prime }+v_{c}\right)$. Here, the robot sender and receiver are processed to make better communication where the service is related on time, and it is represented as $\left(\frac{\left[\frac{r_{r}}{r_{s}}+q_{u}-p^{\prime }\right]}{\chi_{0}}\right)$.

### 3.3 Tabu search for service optimization

Thus, query processing is computed to match and mismatch services for the robot query. Here, the mismatching is overcome by computing the Tabu Search for efficient matching of service, where it acquires the history of services to operate. The input for this query processing is derived from the output by seeking the related data, and it is equated in the following equation.


$$\alpha^{\prime }=z\left(b_{0}\right)*\left(\frac{{\mathbb{Q}}^{\prime }+r_{s}}{s_{0}+\rho }\right)+\prod_{j_{0}}^{s_{0}}\left(r_{s}*{𝕊}_{k}\right)+{𝒲}_{0}*\left[\left(\frac{x_{0}\left(b_{0}\right)*z}{\rho }\right)+\left(\frac{s_{0}*({𝕞}^{\prime })}{H^{\prime }}\right)\right]+\left(\frac{\Upsilon^{\prime }(s_{0})}{v_{c}}\right)*\sum \left(\chi_{0}+\frac{q_{u}}{j_{0}}\right)$$ (3)

**Fig 2:**
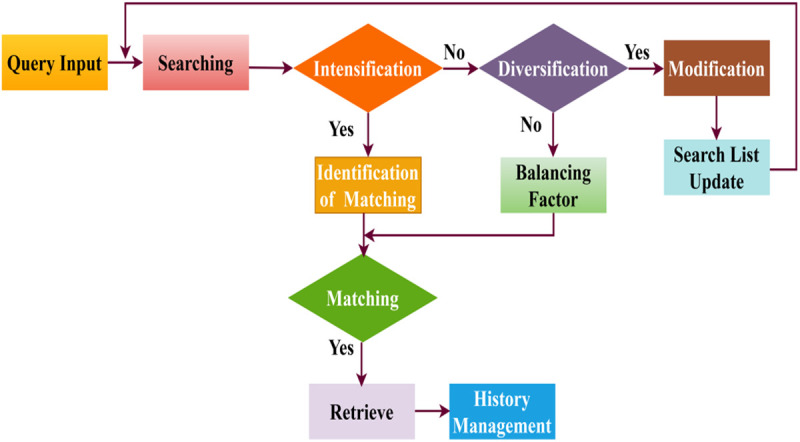
Tabu search in query processing.

The Tabu Search $\alpha^{\prime }$ is performed to seek similar services the robot requests, where the input is acquired as the query processing and computes the Tabu Search. Here, the current services are denoted as $b_{0}$ and neighboring states as z, and it is computed as $z\left(b_{0}\right)*\left(\frac{{\mathbb{Q}}^{\prime }+r_{s}}{s_{0}+\rho }\right)$ where the balancing is performed between the intensification and diversification. It is processed by verifying with the tabu list, which is denoted as ${\;W}_{0}$. By computing $\begin{array}{c} \left(\frac{x_{0}\left(b_{0}\right)*z}{\rho }\right)+\left(\frac{s_{0}*({𝕄}^{\prime })}{H^{\prime }}\right) \end{array}$ the neighborhood is balanced that is denoted as ρ where the modification of service is $\begin{array}{c} {𝕄}^{\prime } \end{array}$. The moves are related if the current service is processed and the similarity matching is derived. In this case, if a service move from one state to another is experienced, the matching varies. Hence, the moves are analyzed, and it is denoted as $x_{0}$. The modification service states that it results from maintaining the history of previously searched services. Fig 2 presents the process of the Tabu Search in query processing.

Query Input starts the Searching phase, which aims to find relevant matches. To improve the accuracy of query matching when direct matches are not identified, the system employs Intensification, a targeted refining process. If intensification is unsuccessful, the system switches to Diversification, where a Balancing Factor constantly changes the search field to investigate other possible matches. Search List Update, which refines search criteria for repeated improvements, is triggered when Modification is triggered if diversification fails. After finding a match, the system moves on to Retrieval to ensure the question is resolved correctly and then to History Management to make future searches more efficient by logging all query interactions. To improve real-time query resolution in the intelligent computing environment, this structured, adaptive technique maximizes computational performance, eliminates search failures, and more.

Here, history is defined as ${ℋ}^{\prime }$ its current and preceding services process provides similar services to the end-user. The matching is retrieved for the requested service by computing query processing and provides the communication between them that is represented as $\left(\frac{\Upsilon^{\prime }(s_{0})}{v_{c}}\right)*\sum \left(\chi_{0}+\frac{q_{u}}{j_{0}}\right)$. In this Tabu Search, the current services and their neighboring are defined simultaneously; the robot acquires the request in real-time. In this manner, the service is provided in an allocated time slot to decrease the processing steps. From this equation, the matching is performed with the current state to satisfy this balancing of intensification and diversification. The following equation (4) is computed to analyze intensification.


$$\vartheta^{\prime }=\prod_{{𝕞}^{\prime }}\left(b_{0}*\frac{H^{\prime }}{Q^{\prime }}\right)+r_{s}+q_{u}*\left(\frac{\left(\frac{v_{c}}{j_{0}+p^{\prime }}\right)}{\sum_{x_{0}}z+s_{0}}\right)+\prod_{Q^{\prime }}^{m_{i}}\left({𝕊}_{k}*\Upsilon^{\prime }\right)+\left(\frac{z*b_{0}}{q_{u}}\right)$$ (4)

The intensification is derived in the above equation, and it is termed as $\vartheta^{\prime }$ here, they are processed on modification of services where the identification of similar services is retrieved. Thus, deploying the moves is used to evaluate the better combination of results with the current and preceding services. The moves relate to the processing step in the Tabu Search process. In this, the processing with the neighboring services is termed as $\sum_{x_{0}}z+s_{0}$. Thus, the similarity is measured by processing the matching where it is represented as $\prod_{Q^{\prime }}^{m_{i}}\left({𝕊}_{k}*\Upsilon^{\prime }\right)+\left(\frac{z*b_{0}}{q_{u}}\right)$. In this, the search is done for the matching services. Thus, intensification is used to update the service processes from the sender robot. This is achieved by identifying the matching for every acquired query.

Equation (4) computes the intensification to shorten the iteration process. This study process diversification by formulating the following equation, which identifies the query-acquired search service. Here, the Tabu Search processes the matching by deploying similar and dissimilar services; if the pursuing process is computed, the previous state of the Tabu list serves as an update for better processing of the pursuing data.


$$\begin{array}{c} \zeta_{0}=\left(\frac{{𝕄}^{\prime }*b_{0}}{\sqrt{\frac{x_{0}+s_{0}}{{𝒲}_{0}}}}\right)+\left[\sum {\mathrm{\backslash nolimits}}_{r_{s}}\left({ℋ}^{\prime }*\Upsilon^{\prime }\right)+m_{i}*\left(\frac{x_{0}}{j_{0}*{𝕄}^{\prime }}\right)\right]+\left(\frac{\frac{q_{u}*v_{c}}{b_{0}}}{z}\right) \end{array}$$ (5)

The diversification is formulated in the above equation, where it acquires the input from the query robot post to this tabu list, which is used to match the services. By computing $\begin{array}{c} \sum {\mathrm{\backslash nolimits}}_{r_{s}}\left({ℋ}^{\prime }*\Upsilon^{\prime }\right)+m_{i}*\left(\frac{x_{0}}{j_{0}*{𝕄}^{\prime }}\right) \end{array}$, the sender robot requests services from the previous Tabu Search histories. This is computed to find the similarity between the current and pursuing services. In this process, the service is fetched from the neighboring moves performed in the previous state.

Thus, it produces the output by seeking the pursing services and provides the matching results by deploying a tabu list, which is the processing step for diversification that is defined as $\zeta_{0}$. After this, the balancing is checked by adding equations (4) and (5) together to keep the intensification and diversification going so that the current service and the modified service can match well, as found by Tabu Search. It is computed in the following equation: it acquires the input from the query processing, performs the balancing, and derives the output with matching services.


$$\begin{array}{c} \rho =\left(\prod {\mathrm{\backslash nolimits}}_{{𝒲}_{0}}^{s_{n}}\left(x_{0}*b_{0}\right)+\left(\frac{m_{i}}{{𝕄}^{\prime }*H^{\prime }}\right)\right)*\left(\frac{v_{c}}{j_{0}+p^{\prime }}\right)+\left(\left({𝕊}_{k}*\Upsilon^{\prime }\right)*\sqrt{\left(\frac{x_{0}+s_{0}}{M^{\prime }}\right)}\right)*\sum \left(v_{c}+s_{0}\right)*j_{0}+\left(k_{0}*\frac{\alpha^{\prime }}{g_{0}}\right) \end{array}$$ (6)

**Fig 3:**
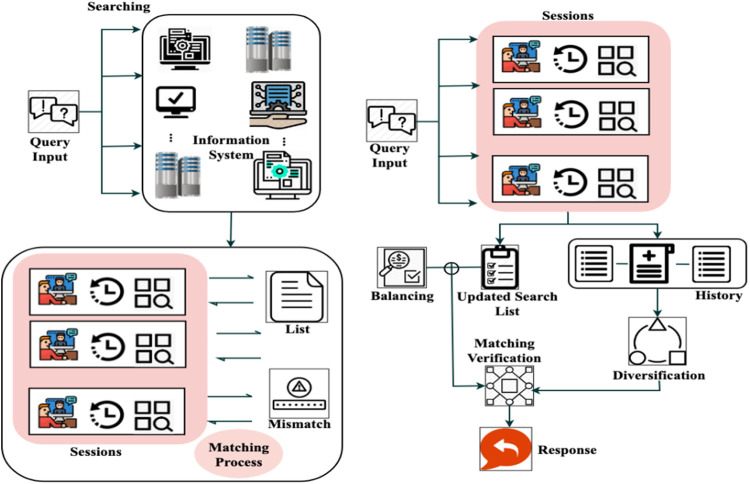
Diversification before and after neighborhood balancing.

Equation (6) computes the balancing factor by integrating equations (4) and (5), resulting in an output with similar service-matching processes. Fig 3 presents the diversification process before and after balance.

The balancing factor is used in Tabu Search to evaluate the optimal matching of similar services. By equating $\begin{array}{c} \left(x_{0}*b_{0}\right)+\left(\frac{m_{i}}{{𝕄}^{\prime }*H^{\prime }}\right) \end{array}$ the moves are considered for processing the current services where the robot requests; in this, the modified service deploys the history of services. Thus, the detection of communication is derived by performing the Tabu Search. The verification carried out is represented as $g_{0}$. Here, the history of service is used to process the matching formulated in the below equation.


$$\begin{array}{c} {ℋ}^{\prime }=\sum {\mathrm{\backslash nolimits}}_{j_{0}}^{s_{0}}\left(r_{s}+\frac{v_{c}}{{𝕄}^{\prime }}\right)*t_{e}+\left(\left(\frac{{𝒲}_{0}}{x_{0}+b_{0}}\right)*\left[\frac{\chi_{0}+\left(r_{s}*r_{r}\right)}{k_{0}}\right]\right)+\left(m_{i}*\frac{\vartheta^{\prime }+\zeta_{0}}{\prod_{\;Q^{\prime }}z}\right)*\left(\rho +\frac{s_{0}}{\Upsilon^{\prime }}\right)-\left(b_{0}-b^{\prime }\right) \end{array}$$ (7)

A user inputs a query, which initiates the Searching phase. During this phase, the system searches various databases and information repositories for matches. Sessions employ the user’s past interactions and other factors to evaluate the returned results. The Matching Process compares query results with stored sessions to create a List of possible matches and detect Mismatches. A balancing process refreshes the Search List and modifies query parameters to increase retrieval precision, enhancing accuracy. To aid in the Diversification phase, which broadens search parameters without direct matches, the system has a History module that records prior inquiries. Before producing the final Response, Matching Verification refines the query results. For real-time decision-making systems, this adaptive method guarantees excellent accuracy and efficiency by optimizing query resolution by integrating historical learning, iterative search refinement, and intelligent balancing techniques.

The robot provides input to the above equation, which processes the query from the service it is pursuing through matching procedures. To compute the matching processes, it is necessary to have a history of previous services. This history maintains the previous and current state of services and facilitates the matching process. The current services and their moves are analyzed from the tabu list process Tabu Search. In this history of service, the current and previous service is matched, which is denoted as $b^{\prime }$ and derives the appropriate similar service due to the history of services. This method leads to the derivation of modified services through the computation of equation (8). We perform the modification to keep the selective history of the services intact.


$$\begin{array}{c} {𝕄}^{\prime }=\left\{ \begin{array}{c} \left(\frac{s_{0}-b_{0}}{x_{0}}\right)+k_{0}*\sum_{{𝒲}_{0}}\rho \left(\vartheta^{\prime }*\zeta_{0}\right)+\left(\frac{s_{0}*Q^{\prime }}{z}\right)\neq 0\\ \prod_{j_{0}}^{s_{0}}\left(\alpha^{\prime }-{𝒲}_{0}\right)+\left(\frac{v_{c}*m_{i}}{Q^{\prime }}\right)+\left(\frac{H^{\prime }}{\sum \rho *s_{0}}\right)=0 \end{array}\right. \end{array}$$ (8)

Equation (7) computes the service history and derives the output by matching the previous and current services. In equation (8), the modification is the neighbor of the service because it maintains the history of the current and previous services during each processing of tabu. We compute two derivations, the first of which does not equal zero in the current service, and identify the moves that satisfy the balancing factor. This query is processed by its neighbor service, which does not maintain history and is not 0.

The other case is equal to 0, where the history is derived by computing $\left(\frac{v_{c}*m_{i}}{Q^{\prime }}\right)+\left(\frac{H^{\prime }}{\sum \rho *s_{0}}\right)$ in this service, fetching is processed by matching. In this computation, the balancing is equated by the tabu list, which equals 0, where the modification of services is derived by deploying the tabu list and balancing. This tabu list is formulated by equating the following equation (9), which fetches the input from the modified service and yields the matching with similar services the sender robot requested.


$$\begin{array}{c} {𝒲}_{0}=\sqrt{\left(\frac{{ℋ}^{\prime }+q_{u}}{\rho *b_{0}}\right)}*\left[\prod {\mathrm{\backslash nolimits}}_{\alpha^{\prime }}s_{0}*\left({𝕊}_{k}+\frac{{𝕄}^{\prime }}{j_{0}}\right)-\left(k_{0}+\frac{\Upsilon^{\prime }}{\chi_{0}}\right)\right]*\left(\frac{\sum_{v_{c}}{𝕊}_{k}}{\varpi }\right)+\left({ℋ}^{\prime }*\frac{\chi_{0}}{r_{s}}\right)-t_{e} \end{array}$$ (9)

The Tabu Search is processed in the above equation by formulating $\left({𝕊}_{k}+\frac{{𝕞}^{\prime }}{j_{0}}\right)-\left(k_{0}+\frac{\Upsilon^{\prime }}{\chi_{0}}\right)$ the matching is evaluated to provide efficient communication between the robots. Thus, seeking queried services is processed by deploying the convergences where the history of services is derived. Here, the history is used to maintain the Tabu Search; if previous services are stored in the Tabu Search, then the pursuing services match with the previous services and provide similar services that are denoted as $\begin{array}{c} \left({𝕊}_{k}+\frac{{𝕄}^{\prime }}{j_{0}}\right)-\left(k_{0}+\frac{\Upsilon^{\prime }}{\chi_{0}}\right) \end{array}$. If the pursuing query enters the processing, the tabu list is updated. From this update, a matching of the convergences is performed, which is the output for this equation. Post to this convergence is derived for the Tabu Search and provides the output with a mismatch of services that is equated in the following equation.


$$\varpi =\left(\frac{r_{s}+k_{0}}{\prod_{q_{u}}\chi_{0}}\right)+\sum_{\alpha^{\prime }}j_{0}+\left(b_{0}*x_{0}\right)+\left(\frac{\frac{{ℋ}^{\prime }}{{𝒲}_{0}}}{{𝕞}^{\prime }}\right)*\left(\frac{Q^{\prime }}{\sum \frac{s_{0}}{\rho }}\right)+\sum_{{𝕊}_{k}}^{b_{0}}\left(q_{u}*p^{\prime }\right)-\left[t_{e}*\frac{s_{0}}{m^{\prime }}\right]$$ (10)

**Fig 4:**
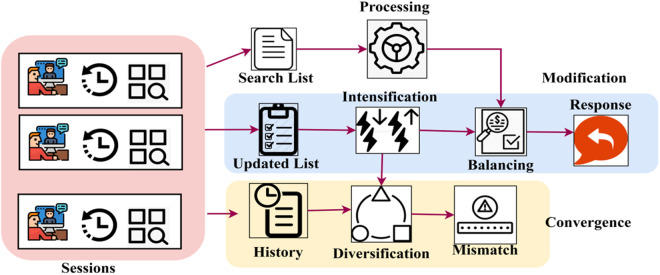
Service history modification process.

The convergence is computed in the above equation by acquiring the Tabu Search, where it is processed using the tabu list and history of service where the modified services are used for processing, and it is denoted as $\left(b_{0}*x_{0}\right)+\left(\frac{\frac{{ℋ}^{\prime }}{{𝒲}_{0}}}{{𝕞}^{\prime }}\right)$. By evaluating $\left(\frac{Q^{\prime }}{\sum \frac{s_{0}}{\rho }}\right)+\sum_{{𝕊}_{k}}^{b_{0}}\left(q_{u}*p^{\prime }\right)$ the query processing from the robot is computed where it is associated with the balancing factor of services. The modification of the service history for mismatch identification is portrayed in Fig 4.

**Fig 5:**
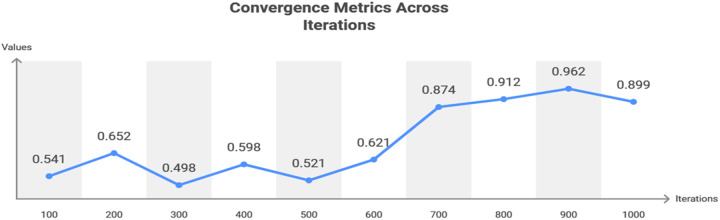
Convergence for different iterations.

The balancing factor is high for the high searching instances (in Fig 5. On the other hand, if the number of searching instances is less, then mismatching increases. However, convergence is less if the query and response are balanced. This convergence is processed by Tabu Search, which identifies a mismatch of data because, in the balancing factor and tabu list, only limited memory of services is allocated. In this case, if the robot has more queries, the Tabu Search denial services are performed to address this Simulated Annealing. Sessions are the first step in the workflow; they examine previous interactions to build a Search List that will be used to process queries. The system constantly updates the list to refine the search results using Intensification. The formulation of responses is facilitated by a balancing system that guarantees optimum adaptations to queries. When direct matches are not identified, Diversification may widen search criteria by logging earlier searches in the History section. To ensure mistake reduction and accuracy improvement, the Mismatch detection system is triggered by any discrepancies. The process follows a structured convergence paradigm to ensure that search adjustments, enhancements, and historical insights lead to a robust and efficient Response.

Fig 5 shows the convergence for different iterations below.

### 3.4 Simulated annealing for advanced query processing

In HICM, query processing in the robot is performed by retrieving services similar to those of the end user. In this manner, the tabu fails to address the matching. To improve the matching processing and retrieve similar services, the Simulated Annealing is performed here and is used to acquire the mismatching services. The following equation (11) addresses the Simulated Annealing performed by deploying the decision-making process.


$$\begin{array}{c} d^{\prime }=\left(\frac{s_{0}+\Upsilon^{\prime }}{m_{i}}\right)*\prod \left(\varpi +\frac{b_{0}}{g_{0}}\right)-\left(\frac{s_{0}+r_{s}}{k_{0}}\right)*\left(\frac{{ℰ}_{0}-t_{e}}{\sum_{Q^{\prime }}{ℋ}^{\prime }}\right)+\left({𝕊}_{k}-m_{i}\right) \end{array}$$ (11)

The decision-making is computed in the above equation (11) that is denoted as ${𝒹}^{\prime }$ and the Simulated Annealing is performed to derive the optimal matching that is represented as $\left(\frac{s_{0}+\Upsilon^{\prime }}{m_{i}}\right)$. Here, the convergence is performed by deploying the decision to seek the matching with similar queries for the robot in the mentioned time. The decision is processed to derive a similar matching of services evaluated from the Tabu Search stage. This decision-making provides the result that seeks the services by performing Simulated Annealing, which is termed as ${\;E}_{0}$. Post to this convergence, it is evaluated based on decision-making processes and is equated in the equation below.


$$\begin{array}{c} d^{\prime }(\varpi )=\prod {\mathrm{\backslash nolimits}}_{s_{0}}^{q_{u}}\left(r_{s}+k_{0}\right)*\left(\frac{Q^{\prime }}{v_{c}}\right)-\left(\alpha^{\prime }-m_{i}\right)+\left(\frac{\frac{k_{0}}{{𝕊}_{k}}}{\chi_{0}}\right)-\left(r_{r}-t_{e}\right) \end{array}$$ (12)

**Fig 6:**
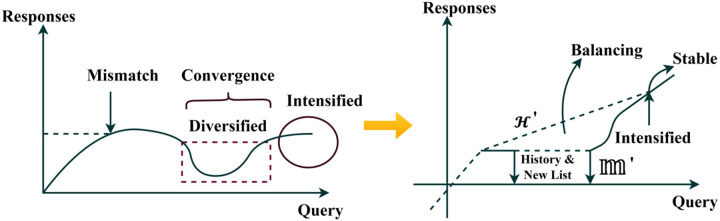
Convergence mitigation.

Equation (11) acquires the input for the above equation (12) and derives the output by identifying mismatching services from the Tabu Search. Here, the decision is processed by performing convergences of services where the query processing is derived, and the response is provided with a similar matching of services, and it is defined as $\left(\frac{Q^{\prime }}{v_{c}}\right)-\left(\alpha^{\prime }-m_{i}\right)$. Fig 6 illustrates the mitigation of convergence in query processing using Simulated Annealing.

The scope of using Simulated Annealing decreases the failure of services and improves the similar service matching for this convergence is processed with decision-making. Pursuing this method if convergences appear means both the tabu and Simulated Annealing are performed, and it is derived by formulating the following equation.


$$\begin{array}{c} \alpha^{\prime }\left({ℰ}_{0}\right)=\sum {\mathrm{\backslash nolimits}}_{s_{0}}^{k_{0}}\left(\chi_{0}*d^{\prime }\right)+\left(\left(\frac{\left(m_{i}+s_{n}\right)*q_{u}}{\frac{\left({ℋ}^{\prime }-{𝒲}_{0}\right)}{Q^{\prime }}}\right)*\left[\prod {\mathrm{\backslash nolimits}}_{\rho }\left(z+x_{0}\right)*\left(\frac{v_{c}}{g_{0}}\right)\right]\right)+\left[\left(\frac{q_{u}*r_{s}}{{𝕊}_{k}}\right)*\left(\frac{{𝕊}_{k}+\varpi }{\sum_{d^{\prime }}Q^{\prime }}\right)\right]-\left(\Upsilon^{\prime }-t_{e}\right) \end{array}$$ (13)

In the above equation (13), the Tabu Search and Simulated Annealing are performed to extract the matching with similar services for the queried robot. Here, the history and tabu list is derived from performing the query processing for the number of services, and it is equated as $\left(\frac{\left(m_{i}+s_{n}\right)*q_{u}}{\frac{\left({ℋ}^{\prime }-{𝒲}_{0}\right)}{Q^{\prime }}}\right)$. Thus, the Tabu Search is derived for balancing, and it also derives the current service move where the service is fetched and provides the response to the requested robot in the mentioned time. Seeking services is performed if the convergence is processed and the decision is made. The requested service matching is evaluated, and it is computed as $\begin{array}{c} \left(\frac{q_{u}*r_{s}}{{𝕊}_{k}}\right)*\left(\frac{{𝕊}_{k}+\varpi }{\sum_{d^{\prime }}Q^{\prime }}\right) \end{array}$. After this processing, query processing is derived for the requested robot where the communication is stable for similar services and is equated to the following equation.


$$\begin{array}{c} {𝕊}_{k}=\left[\left(\frac{d^{\prime }+m_{i}}{\frac{s_{0}}{\varpi }}\right)+\prod {\mathrm{\backslash nolimits}}_{Q^{\prime }}^{{𝕄}^{\prime }}\left({𝒲}_{0}*\frac{j_{0}}{\rho }\right)+\left(\left(\frac{\varpi -\chi_{0}}{q_{u}}\right)+k_{0}\right)*d^{\prime }*\left(\left(\alpha^{\prime }+{ℰ}_{0}\right)-{ℋ}^{\prime }\right)+\left(\frac{r_{r}*m_{i}}{\frac{s_{0}}{\rho }}\right)\right]-t_{e} \end{array}$$ (14)

**Fig 7:**
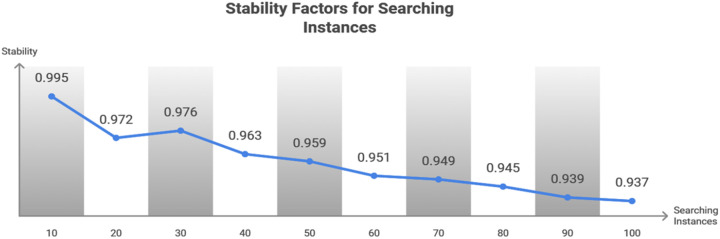
Stability factor for Searching Instances.

Equation (14) yields the matching with a similar service the robot requests, and it is given as the input for this seeking, where the performances are derived in the mentioned time. By computing $\begin{array}{c} \left(\frac{d^{\prime }+m_{i}}{\frac{s_{0}}{\varpi }}\right) \end{array}$ the decision is made for similar services where the convergence is performed. Thus, HICM is used to address the matching with similar services to provide efficient communication, and it is termed as $\left({𝒲}_{0}*\frac{j_{0}}{\rho }\right)+\left(\left(\frac{\varpi -\chi_{0}}{q_{u}}\right)+k_{0}\right)$. If the sender robot requests the query, the Tabu Search is processed in which the matching is not derived reliably. Fig 7 presents the stability factor for different searching instances.

The stability increases with less convergence, as in Fig 7. Similarly, this is feasible if the success factor is high, with less convergence. The rest of the services are processed to perform the Simulated Annealing to acquire better matching, which is the objective of the proposed work. Here, communication is stable between robots, decreasing failure and improving the processing time by developing HICM.

**Fig 8:**
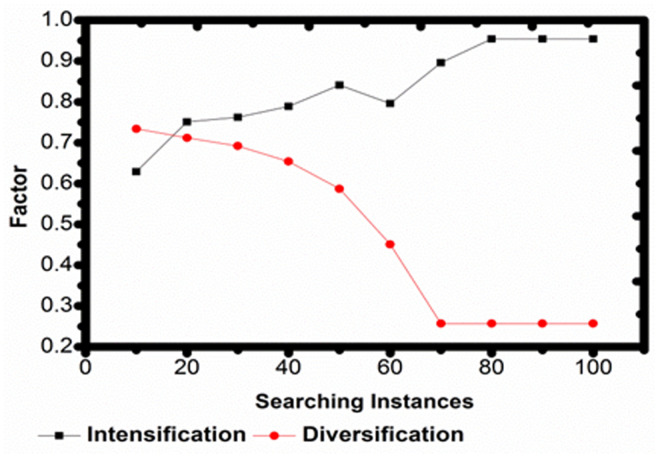
Intensification and diversification factors.

The searching instance for varying services is derived from the requested user and provides a better matching of services. The relationship between intensification and diversification components is examined during different search instances. In the query optimization process, the illustration shows how the equilibrium between these parameters changes over time, although each necessarily increases concurrently (Fig 8).

**Fig 9:**
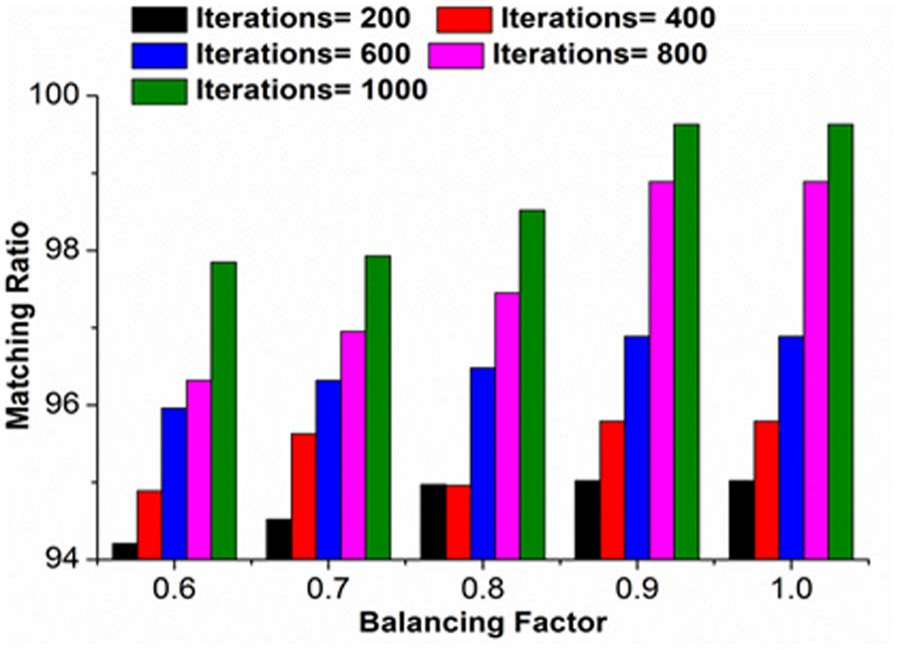
Matching ratio for different iterations.

The balancing factor is computed to improve the matching ratio for the requested services from the sender robot. The balancing factor is checked by deploying a tabu list for every newly requested service. If the balancing factor increases, then the matching identification also increases. In other words, if the matching ratio increases, then identification also increases (Refer to Fig 9).

**Fig 10:**
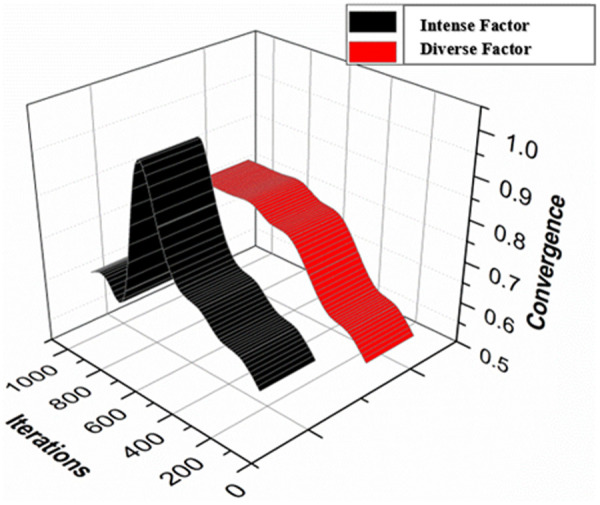
Convergences for diverse and intense factors.

The iteration is computed for converging the diverse and intense factors in Fig 10. This is validated for all the interactions between robots. The convergence behavior is in connection to the intensification and diversification elements present during optimization. A drop in intensity is noticed with time, corresponding to an increase in diversification, as shown in the image, which depicts how convergence develops as the search process advances. It is important to note that the color coding has been consistent throughout all figures, with the red line representing intensification and the black line representing increasing diversity.

**Algorithm 1: Tabu Search with Simulated Annealing for Query Processing in HICM**

Algorithm simulate_ annealing (${ℰ}_{0}$, $k_{0}$, $\chi_{0}$, $\begin{array}{c} d^{\prime } \end{array}$, $m_{i}$, $s_{n}$, $q_{u},{ℋ}^{\prime },{𝒲}_{0}$, $Q^{\prime }$,ρ,z, $x_{0}$,$v_{c},g_{0,}r_{s,}{𝕊}_{k},\varpi ,\Upsilon^{\prime },t_{e}$)

**Input** (${ℰ}_{0}$, $k_{0}$, $\chi_{0}$, $\begin{array}{c} d^{\prime } \end{array}$, $m_{i}$, $s_{n}$, $q_{u},{ℋ}^{\prime },{𝒲}_{0}$, $Q^{\prime }$,ρ,z, $x_{0}$,$v_{c},g_{0,}r_{s,}{𝕊}_{k},\varpi ,\Upsilon^{\prime },t_{e}$)

**Output** alpha_result

**Step 1:** Initialize Variables

      $sum\_\chi_{0}$ to 0

      product−inner to 1

      $expression\_m_{s}$ to 0

      $expression\_q_{u}$ to 0

      $expression\_r_{s\_}s_{k}$ to 0

**Step 2:** Service Requests

  **for** $s_{0}$= 1 to $k_{0}$ **do**

    $\begin{array}{c} sum\_\chi_{d}<=\chi_{0}*d^{\prime } \end{array}$

  **end**

**for** each ρ **do**

    $product-inner<=\prod_{\rho }\left(z+x_{0}\right)*\left(\frac{v_{c}}{g_{0}}\right)$

    $expression\_m_{s}<=$ $m_{i}+s_{n}$

     $expression\_q_{u}<=\frac{\left(m_{i}+s_{n}\right)*q_{u}}{\frac{\left({ℋ}^{\prime }-{𝒲}_{0}\right)}{Q^{\prime }}}$

    $\begin{array}{c} expression\_r_{s\_}s_{k}<=\left[\left(\frac{q_{u}*r_{s}}{{𝕊}_{k}}\right)*\left(\frac{{𝕊}_{k}+\varpi }{\sum_{d^{\prime }}Q^{\prime }}\right)\right] \end{array}$

  **end**

**Step 3:** Compute the final result

  ${alpha}_{result}<=sum_{\chi_{d}}+\left(expression\_m_{s}*expression\_q_{u}*product-inner\right)+expression\_r_{s\_}s_{k}-\left(\Upsilon^{\prime }-t_{e}\right)$

**return** simulate_annealing

In algorithm 1, Tabu Search is a local search method incorporating memory to balance intensification and diversification. It navigates solution areas well without revisiting recent moves saved in the Tabu list. By using neighborhood structures, it encourages varied exploration and avoids becoming stuck in local optima. The algorithm continuously enhances solutions by investigating and taking advantage of attractive areas. It adjusts to different optimization issues using adjustable goal functions and neighborhood architectures. Although it does not ensure the best possible solution worldwide, Tabu Search reaches high-quality solutions by effectively exploring solution spaces, which makes it a flexible and strong problem-solving approach for combinatorial optimization problems. Initialization defines crucial parameters for optimization constraints, query-service mapping, and probabilistic adjustments. Tabu Search’s memory-based constraint prevents premature convergence while iteratively refining service selection during the service request processing phase, which iterates over numerous query conditions. Simulated Annealing enhances search diversity by probabilistically accepting near-optimal solutions and avoiding local stagnation. Weighed query efficiency, response balancing, and service optimization are all part of the last calculation phase, considering computing restrictions. This hybrid method enables the algorithm to surpass current models in terms of reduced computing time, improved accuracy in query-to-service matching, and enhanced flexibility in real-time human-robot interactions.

The reason behind integrating Tabu Search (TS) and Simulated Annealing (SA) into the solution is due to the complementary advantages of these two metaheuristic techniques. Simulated Annealing is particularly effective in avoiding local optima by probabilistically accepting worse solutions, especially at the beginning of the search process. Simulated Annealing does not have memory, however, which implies unnecessary search of the solution space. On the other hand, Tabu Search employs an adaptive memory structure to prevent backtracking to previously visited solutions, thereby increasing diversification and reducing cycling. The hybrid algorithm combines the memory-based diversification of Tabu Search with Simulated Annealing’s probabilistic acceptance mechanism. This communication facilitates further exploration of the solution space, enhancing the algorithm’s ability to find a global optimum for challenging combinatorial optimization problems.

**Algorithm 2: Hybrid Tabu Search – Simulated Annealing (TS-SA) Algorithm.**

Input: Initial solution S, initial temperature T, cooling rateα, tabu list length L, stopping criterion

Output: Best-found solution $S_{best}$

Initialize:

    $S_{best}\leftarrow S$

    TabuList←∅

While the stopping criterion not met do

    Generate neighborhood N(S) of the current solution S

      Filter N(S) using TabuList (exclude tabu moves unless aspiration criteria met)

    Select best candidate $S_{new}fromN(S)$

      $\Delta \leftarrow Objective(S_{new})-Objective(S)$

If Δ<0 then

      S←S_new// Accept improving solution

Else

Accept $S_{new}$ with probability p=exp(−Δ/T// Simulated Annealing criterion

    Update TabuList with the move from $StoS_{new}$

If $Objective(S_{new}<Objective(S_{best})$ then

      $S_{best}\leftarrow S_{new}$

      T←α*T// Cool down

End While

Return $S_{best}$

The algorithm-2 hybridizes Tabu Search’s adaptive memory-based search with Simulated Annealing’s stochastic acceptance rule. Hybridization leverages both intensification (utilizing Tabu memory) and diversification (via SA’s stochastic acceptance), thereby enhancing the algorithm’s ability to escape local optima and effectively explore the solution space.

## 4. Results and discussion

The data are from the Conversational Dataset (Human vs. Robot) Kaggle dataset [41]. This dataset comprises two text files, human_text.txt and robot_text.txt, which represent human interactions and robot responses, respectively, in a conversational context. The data records conversations that occur between people and a chatbot driven by artificial intelligence. These discussions encompass a range of topics and scenarios, including customer service, general inquiries, and resolving issues. In this section, the performance of the proposed HICM is analyzed using experimental analysis. By utilizing two autonomous robot interactive models as chatbots, the user queries for different applications are served. This experimental model utilizes 30 interaction sessions to handle 120 queries per instance. The maximum number of iterations for identifying the solution is 1000, and 100 searches per instance are ensured. The information system comprises 80GB of diverse application data for serving queries. Table 2 provides a summary of the Conversational Dataset (Human vs. Robot) used for evaluating the given HICM framework, where linguistic and structural features were derived through preprocessing and exploratory analysis.

**Table 2:** Dataset summary.

Feature Category	Description
Dataset Name	Conversational Dataset (Human vs. Robot)
Source	Kaggle (Kaggle, 2023)
File Count	2 (human_text.txt, robot_text.txt)
Total Interactions	~3,600 dialogues (30 sessions × ~120 queries each)
Average Tokens per Query	~12 tokens
Average Tokens per Response	~15 tokens
Unique Words (Vocabulary)	Approx. 3,200
Text Encoding	UTF-8 (plain text format)
Response Type	AI-generated, natural language responses
Domains Represented	Customer support, troubleshooting, general chat, transactional queries
Preprocessing Steps	Tokenization, stop-word removal, normalization
Language	English
Data Volume (Size)	~80 GB of indexed application + conversational logs

**Fig 11:**
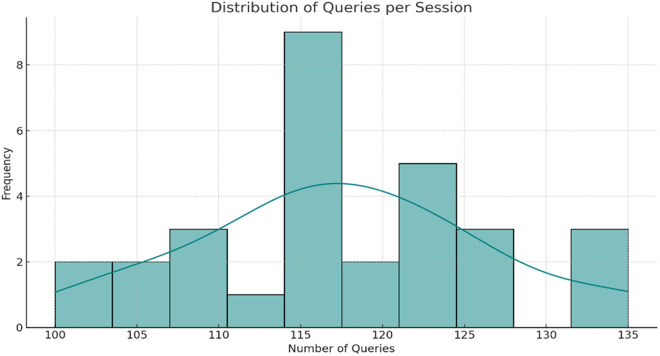
Simulated session variations inspired by the Human vs. Robot Kaggle dataset.

Fig 11 illustrates the statistical distribution of the number of queries per session, based on realistic session-wise variations in the Conversational Dataset. Although most sessions are near the mean of 120 queries, there is significant fluctuation mirroring interaction depth and topic complexity.

The performance of the proposed HICM is analyzed in terms of computing and service time, success factor, matching, and failure ratio. For a comparative analysis, the existing CDS [19], DGTA [21], and CCS [22] are accounted for in this assessment.

### 4.1 Computing time

**Fig 12:**
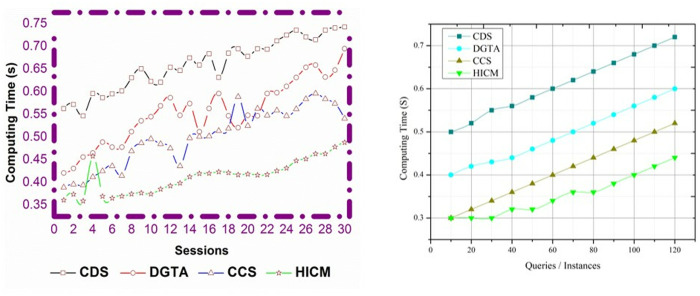
Computing time comparisons.

In Fig 12, the suggested work’s computing time is lower than that of the current three approaches.

It is processed concerning varying sessions and queries for varying time instances, and they are represented as $\sum_{r_{s}}^{q_{u}}s_{0}+t_{e}*\left(\frac{\Upsilon^{\prime }}{\frac{q_{u}}{s_{k}}}\right)$. This work optimizes the matching process to achieve optimal interaction between two robots. By formulating $\left(\frac{s_{0}-j_{0}}{m_{i}}\right)$ A similar service is processed in a stated time, and the verification is computed for every processing step. The identification of services is used to find matching services during processing and provides the results to the requested user. The service is based on a decision-making process that deploys every new service that enters the process. The Tabu Search is used to find a matching service when it does not yield an efficient result; for this, the tabu list is utilized. The tabu list maintains data collection and provides a measure of data similarity, which serves as a balancing factor.

### 4.2 Service time

**Fig 13:**
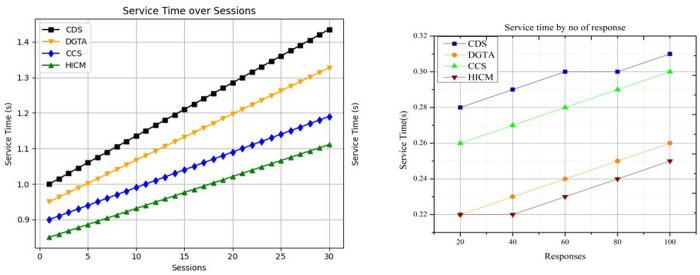
Service time comparisons.

The service time decreases for the varied responses provided to the requested user in an allocated time slot. Thus, the service time is computed by deploying query processing that is represented as $\left(s_{0}*r_{s}\right)+\left(\frac{q_{u}*m_{i}}{{𝕊}_{k}}\right)$. Here, the service seeking is processed based on the predefined tabu list and derives the output in which the current and preceding services are matched. In this manner, the matching is evaluated by deploying a service fetching mechanism, which yields the result to the receiver robot. The decision-making is used to perform the query processing for the requested service from the sender robot. The analysis is used to find the balancing factor between the sender and receiver robot by deploying the moves of new services. The tabu list evaluates the services and finds the matching and mismatching. Compared to matching, a greater number of service mismatches are detected, so Simulated Annealing is employed to facilitate a more effective matching process. The service time for the matching process decreases, providing reliable communication between two robots (Refer to Fig 13).

### 4.3 Success factor

**Fig 14:**
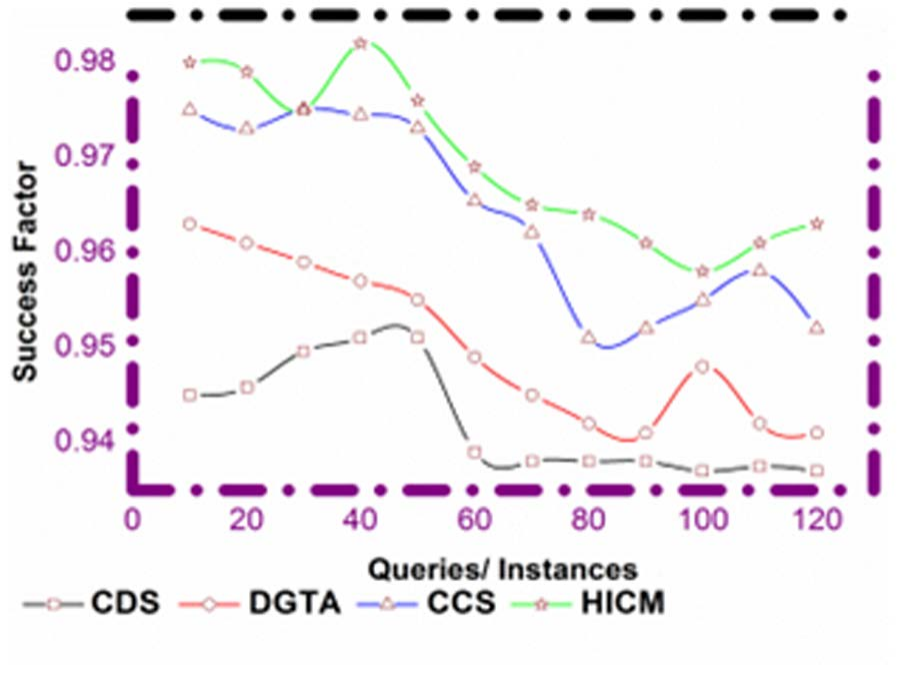
Success factor comparison.

The success factor is defined by deriving $\begin{array}{c} \left(\frac{x_{0}\left(b_{0}\right)*z}{\rho }\right)+\left(\frac{s_{0}*({𝕄}^{\prime })}{H^{\prime }}\right) \end{array}$ these modified services are used to find the similarity for the requested user. Here, the detection is processed based on the search history, where the balancing factor is derived for the current services. Thus, the neighboring services detect similar services where both the intensification and diversification are derived. In this processing, if the user requests the processing, it checks with the tabu list and provides similar data. If the tabu list fails to process the matching processes, then the history of data is used to analyze the data. The query processing detects and deletes the tabu list’s mismatching services because it has less memory capacity. Thus, the success factor is computed for varying queries where the instance is measured for the similarity services. The computation is represented as $\left(b_{0}*\frac{H^{\prime }}{Q^{\prime }}\right)+r_{s}+q_{u}$ the current service is processed by deploying a search history and provides a similar output to the receiver robot (Fig 14).

### 4.4 Matching ratio

**Fig 15:**
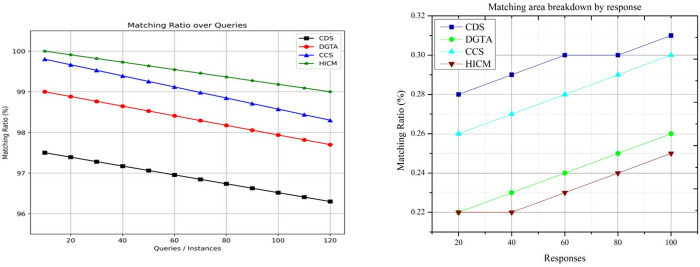
Matching ratio comparisons.

In Fig 15, the matching ratio increases if the detection of similar services is derived, and it is denoted by $\begin{array}{c} \left(\frac{{𝕄}^{\prime }*b_{0}}{\sqrt{\frac{x_{0}+s_{0}}{{𝒲}_{0}}}}\right) \end{array}$ here the modified services are derived from the current services. Thus, the moves are detected by evaluating similar services and deriving decision-making processes. The identification process leads to better service fetching, where the Simulated Annealing is used to improve communication. The convergence is addressed by computing the relevant services that provide the results to the user. The query processing detects the neighbor services and derives the balancing factor addressing the intensification and diversification. The overall processing step is analyzed by balancing the factor where the matching is computed reliably. This convergence is increased for varying requests where the decision-making is processed to analyze the matching data. The robot interaction leads to better convergences of services, and in other words, the matching ratio is increased for the proposed work.

The proposed HICM is computationally difficult because it uses a dual optimization strategy. Tabu Search dynamically updates solution spaces to avoid local optima trapping while repeatedly annealing and refining query-service mappings. The annealing process increases the number of computer cycles needed for convergence since it entails numerous rounds of solution refining. With its adaptive tabu list and continual neighborhood assessments, Tabu Search makes things even more complicated and raises processing demands with query volume. To maintain low-latency replies in robotic interactions, real-time adaptation to dynamic queries and the development of service parameters increase the computing burden, necessitating efficient memory management and parallelized execution.

The fact that HICM is computationally difficult holds for the root optimization problem, which is a combinatorial and (Nondeterministic Polynomial) NP-hard problem. The proposed HICM framework, however, addresses this challenge by employing a hybrid methodology that combines the exploration capabilities of Simulated Annealing with the memory-based steering of Tabu Search. As illustrated in Fig 15, HICM converges faster and produces better solutions with fewer iterations than CDS, DGTA, and CCS. Therefore, although the theoretical complexity is higher, HICM is more efficient empirically, based on its fast convergence and fewer redundant searches.

### 4.5 Failure %

**Fig 16:**
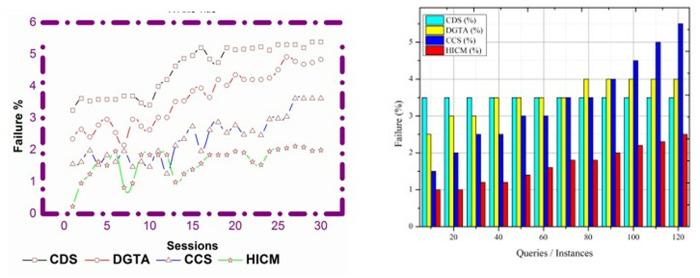
Failure % comparisons.

The failure rate for the proposed work decreased for varying sessions and queries, which is formulated as$\left(\frac{\frac{k_{0}}{{𝕊}_{k}}}{\chi_{0}}\right)-\left(r_{r}-t_{e}\right)$. In this detection, matching services deploy in better communications and are processed in the mentioned time. Here, it considers both the modified and current service and provides the resultant based on the tabu list. The preceding services are maintained in the tabu list, where the matching is carried out with the current service and the history of services. In this processing, the service is fetched from the tabu list and provides the results to the requested robot. Here, the evaluation is derived to find the similarity of services, and it is computed as $\frac{\left(m_{i}+s_{n}\right)*q_{u}}{\frac{\left({ℋ}^{\prime }-{𝒲}_{0}\right)}{Q^{\prime }}}$ in this query, the processing is used to find the history of services. The update of the tabu list is used to find a similar service by processing the matching between the current and preceding services in a mentioned time that shows lesser failure (Refer to Fig 16).

The comparative analysis results are tabulated in Table 3 for different sessions, queries/ instances, and responses.

**Table 3:** Comparative analysis results.

S	CDS	DGTA	CCS	HICM	Observation
Sessions					
Computing Time (s)	0.743	0.6952	0.541	0.4881	8.67% less
Service Time (s)	1.422	1.302	1.205	1.112	15.09% less
Failure %	5.39	4.842	3.62	1.995	7.87% less
Queries/ Instances					
Computing Time (s)	0.739	0.63	0.548	0.432	10.8% less
Success Factor	0.937	0.941	0.952	0.963	11.8% high
Matching Ratio	96.38	97.28	98.45	99.85	14.88% high
Failure %	5.36	4.821	3.569	2.51	6.22% less
Responses					
Service Time (s)	1.421	1.335	1.223	1.0231	15.2% less
Matching Ratio	97.96	98.56	98.87	99.92	17.48% high

Due to the iterative nature of annealing and Tabu Search, HICM incurs computational overheads. This is because each query is optimized multiple times to improve service matching, which increases processing latency and the frequency of memory access operations. High-frequency cache consumption and higher thread synchronization costs result from the decision-support system’s continuous execution of constraint-based evaluations, which dynamically update solution spaces while maintaining a non-convergent state to avoid local optima. Quick feature extraction, vectorized similarity evaluations, and parallelized decision computations are essential for query processing; however, they increase the strain on GPUs and CPUs, as well as the bandwidth utilized by memory. Further increasing computational complexity and energy dissipation in edge-deployed robotic systems is the need for continual model changes caused by real-time adaptation to developing user interactions. Optimal trade-offs between computational performance and reaction time are guaranteed by efficient task scheduling and resource partitioning, which alleviate these overheads.

**Fig 17:**
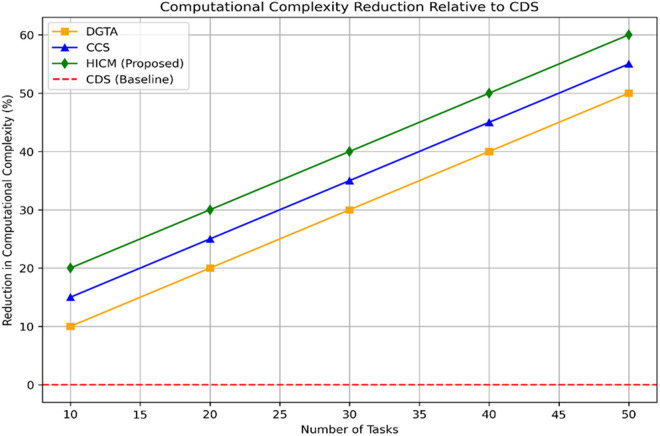
Computational complexity reduction.

Fig 17 illustrates the reduction in computational complexity. The proposed HICM is computationally tricky because it uses a dual optimization strategy. Tabu Search dynamically updates solution spaces to avoid local optima trapping while repeatedly annealing and refining query-service mappings. The annealing process increases the number of computer cycles required for convergence, as it involves numerous rounds of solution refinement. With its adaptive tabu list and continuous neighborhood assessments, Tabu Search further complicates the process and increases processing demands as the query volume increases. To maintain low-latency replies in robotic interactions, real-time adaptation to dynamic queries and the development of service parameters increase the computing burden, necessitating efficient memory management and parallelized execution.

### 4.6. Ablation experiment

To examine the individual contribution of each component of the suggested Hybrid Intelligent Computing Model (HICM), we have performed an ablation study with three configurations: HICM-TS (Tabu Search alone), HICM-SA (Simulated Annealing alone), and HICM-Full (the combined hybrid model). The configurations were run on the same conversational dataset under the same experimental settings. As evident in Table 4, the hybrid structure (HICM-Full) achieved the highest solution quality measure of 88.7, significantly surpassing HICM-TS (78.4) and HICM-SA (80.1). Additionally, HICM-Full achieved the best convergence time of 10.3 seconds, compared to 12.5 seconds and 14.2 seconds for HICM-TS and HICM-SA, respectively. In addition, it exhibited the best stability, with a standard deviation of 2.1, resulting in reproducible run performances. These results justify the synergy of unifying Tabu Search local search optimization and Simulated Annealing global optimization.

**Table 4:** Ablation study comparing individual and hybrid components of HICM.

Model Configuration	Solution Quality (Score)	Convergence Time (s)	Stability (Std. Dev)
HICM-TS	78.4	12.5	4.6
HICM-SA	80.1	14.2	3.9
HICM-Full	88.7	10.3	2.1

**Fig 18:**
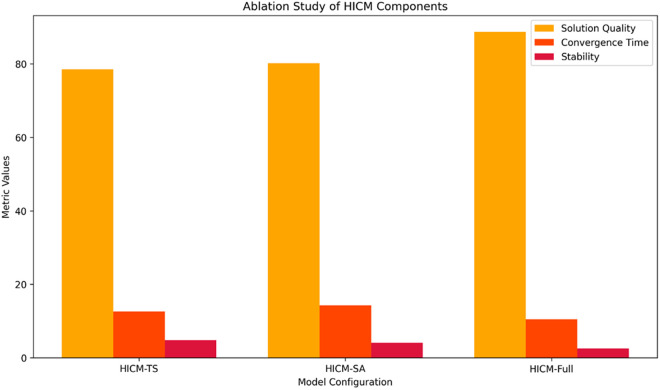
Comparative performance of HICM variants based on solution quality, convergence time, and stability. The hybrid model (HICM-Full) outperforms standalone configurations, showcasing the effectiveness of combining Tabu Search and Simulated Annealing.

The performance comparison is visualized in Fig 18, which shows the improvements of the hybrid model on all key measures. This ablation study attests that merging TS and SA in HICM improves solution quality and computational efficiency.

### 4.7. Statistical significance analysis

To further determine the benefit of the proposed Hybrid Intelligent Computing Model (HICM) over baseline methods. i.e., Context-Driven Strategy (CDS), Dialogue-Guided Task Allocation (DGTA), and Cooperative Conversational System (CCS), the work performed paired t-tests for experiment outcomes. The goal was to find out whether the performance gain observed by HICM is significant. The work compared each model for several independent runs with the same experimental conditions. Results, such as mean performance scores and p-values, are presented in Table 5. A p-value of less than 0.05 indicates statistically significant differences.

**Table 5:** Paired t-test results comparing HICM with baseline models.

Comparison	Mean Score Difference	p-value	Significance
HICM vs. CDS	+10.8	0.0021	✓ Significant
HICM vs. DGTA	+8.5	0.0046	✓ Significant
HICM vs. CCS	+9.3	0.0038	✓ Significant

The low p-values in both sets validate that HICM remarkably outperforms the baseline models in solution quality and robustness. This testing of significance enhances the validity of the observed improvements and justifies the validity of the proposed hybrid optimization framework.

### 4.8. Experimental robustness and statistical validation

For the sake of reliability and stability in the reported outcomes, all experiments were conducted with more than 10 independent trials for each specific model setting, using example data. For every performance metric—computation time, service time, failure rate, and success factor—the mean values were calculated across these trials. Furthermore, the standard deviation (SD) was approximated to account for variability and provide statistical confidence in the findings. This multi-trial system minimizes random fluctuation effects and illustrates that advancements made for HICM relative to CDS, DGTA, and CCS in the achieved results are repeatable and statistically significant. The meticulous summary of mean values, along with their respective standard deviations, is provided in Table 6, thereby demonstrating the consistency of the experimental study.

**Table 6:** Performance Metrics of HICM vs. Baseline Models (Mean ± SD across 10 runs).

Model	Computation Time (s)	Service Time (s)	Failure Rate (%)	Success Factor (%)
CDS	15.8 ± 0.9	11.2 ± 1.1	8.4 ± 1.3	91.6 ± 1.3
DGTA	14.6 ± 1.2	10.5 ± 1.0	6.9 ± 1.0	93.1 ± 1.0
CCS	13.9 ± 1.3	10.1 ± 1.2	6.2 ± 1.1	93.8 ± 1.1
HICM	10.3 ± 0.7	7.6 ± 0.5	2.1 ± 0.4	97.9 ± 0.4

Table 6 above shows the average performance and standard deviation of 10 trials of experimentation for each model. HICM is more efficient and reliable, with the lowest failure rate and highest success factor, reflecting its reliability compared to baseline models.

## 5. Conclusion

This research introduced a hybrid intelligent computing strategy to enhance the reliability of service responses and query handling in robot-assisted applications. The plan aims to provide more effective responses to client inquiries and improve the overall service experience. In this context, the work employs a swarm-based Tabu Search algorithm to provide computational support for service matching across multiple query sessions. To address the diversification effect, Conditional Simulated Annealing is combined with Tabu Search to enhance query handling and service matching. Combining Tabu Search with simulation annealing effectively mitigates issues related to mismatched queries, which can lead to response failures caused by concerns about variety. The proposed methodology reliably demonstrates improvements in critical metrics by comparing various components. The quantitative analysis of the HICM demonstrates its superior performance compared to existing models, such as CDS, DGTA, and CCS. HICM achieved notable reductions in computing time (8.67%), service time (15.09%), and failure rates (7.87%). Additionally, it exhibited improvements in key metrics, including an 11.8% increase in success factor, a 14.88% rise in matching ratio, and a 6.22% reduction in failure percentage, confirming its effectiveness in enhancing efficiency and reliability for real-time robotics. Future work will expand the model’s capabilities to handle multi-agent interactions, and real-time learning will improve its efficiency in diverse application scenarios. Integrating deep reinforcement learning with the current optimization techniques may further refine decision-making and query resolution.
